# c-Fos immunoreactivity in prefrontal, basal ganglia and limbic areas of the rat brain after central and peripheral administration of ethanol and its metabolite acetaldehyde

**DOI:** 10.3389/fnbeh.2013.00048

**Published:** 2013-05-24

**Authors:** Kristen N. Segovia, Regina Vontell, Laura López-Cruz, John D. Salamone, Mercè Correa

**Affiliations:** ^1^Department of Psychology, University of ConnecticutStorrs, CT, USA; ^2^Departament de Psicobiologia, Universitat Jaume ICastelló, Spain

**Keywords:** alcohol, metabolism, early gene, nucleus accumbens, dopamine

## Abstract

Considerable evidence indicates that the metabolite of ethanol (EtOH), acetaldehyde, is biologically active. Acetaldehyde can be formed from EtOH peripherally mainly by alcohol dehydrogenase (ADH), and also centrally by catalase. EtOH and acetaldehyde show differences in their behavioral effects depending upon the route of administration. In terms of their effects on motor activity and motivated behaviors, when administered peripherally acetaldehyde tends to be more potent than EtOH but shows very similar potency administered centrally. Since dopamine (DA) rich areas have an important role in regulating both motor activity and motivation, the present studies were undertaken to compare the effects of central (intraventricular, ICV) and peripheral (intraperitoneal, IP) administration of EtOH and acetaldehyde on a cellular marker of brain activity, c-Fos immunoreactivity, in DA innervated areas. Male Sprague-Dawley rats received an IP injection of vehicle, EtOH (0.5 or 2.5 g/kg) or acetaldehyde (0.1 or 0.5 g/kg) or an ICV injection of vehicle, EtOH or acetaldehyde (2.8 or 14.0 μmoles). IP administration of EtOH minimally induced c-Fos in some regions of the prefrontal cortex and basal ganglia, mainly at the low dose (0.5 g/kg), while IP acetaldehyde induced c-Fos in virtually all the structures studied at both doses. Acetaldehyde administered centrally increased c-Fos in all areas studied, a pattern that was very similar to EtOH. Thus, IP administered acetaldehyde was more efficacious than EtOH at inducing c-Fos expression. However, the general pattern of c-Fos induction promoted by ICV EtOH and acetaldehyde was similar. These results are consistent with the pattern observed in behavioral studies in which both substances produced the same magnitude of effect when injected centrally, and produced differences in potency after peripheral administration.

## Introduction

Ethanol (EtOH) is converted into acetaldehyde in many organs by the enzyme alcohol dehydrogenase (ADH) (Cohen et al., 1980). Acetaldehyde is then metabolized to acetate by aldehyde dehydrogenase (ALDH) (Svanas and Weiner, 1985; Deng and Deitrich, 2008). EtOH crosses the blood brain barrier and is found in peripheral organs as well as in the brain (Eriksson and Sippel, 1977; Deitrich, 1987; Zimatkin, 1991). However, acetaldehyde cannot easily cross into the brain because of the abundance of ALDH in capillary endothelium and surrounding astrocytes of the blood brain barrier (Sippel, 1974; Westcott et al., 1980; Zimatkin, 1991). Only when blood acetaldehyde levels are raised after ALDH blockade, significant amounts of acetaldehyde cross into the brain. In addition, an alternative source of acetaldehyde in the brain is the local intracerebral metabolism of EtOH by the enzyme catalase (Cohen et al., 1980; Aragon et al., 1992; Correa et al., 2012).

EtOH and acetaldehyde do not always have the same pattern of effects on behavior (for a review see Correa et al., 2012). For instance, in rats they produce similar effects on motor activities such as locomotion, and on motivated behaviors such as lever pressing for food in different reinforcement conditions; both exert suppressant effects when peripherally administered (Chuck et al., 2006; McLaughlin et al., 2008), and activating effects when administered in the brain (Arizzi et al., 2003; Correa et al., 2003a,b, 2009a; Arizzi-LaFrance et al., 2006; McLaughlin et al., 2008; Pastor and Aragon, 2008). Yet while the relative efficacy and potency of both substances is very similar after central administration, they are very different after peripheral administration to both rats and mice (Correa et al., 2004, 2009b; Tambour et al., 2005; Chuck et al., 2006; McLaughlin et al., 2008; Closon et al., 2009; Escrig et al., 2012); peripherally acetaldehyde seems always more potent than EtOH.

The induction of Fos/Jun family transcription factors has been widely used as a tool to show neuronal activation in response to a wide range of stimuli (Curran and Morgan, 1995). EtOH exposure through different routes of administration induces early-gene protein expression in several brain regions (Chang et al., 1995; Ogilvie et al., 1998; Bachtell et al., 1999, 2002; Thiele et al., 2000; Knapp et al., 2001; Crankshaw et al., 2003; Canales, 2004), and such expression reflects specific activation of intracellular pathways (Curran and Morgan, 1995; Thiele et al., 2000; Ibba et al., 2009). For instance, c-Fos protein expression is modulated after dopamine (DA) receptor signaling in neurons receiving DA input (Moratalla et al., 1992; Nguyen et al., 1992; Farrar et al., 2010; Pardo et al., 2012, 2013; Segovia et al., 2012). EtOH, as well as acetaldehyde, have been demonstrated to regulate DA release in some of these areas (Di Chiara and Imperato, 1986; Acquas et al., 1993; Melis et al., 2007; Bustamante et al., 2008; Enrico et al., 2009; Sirca et al., 2011). However, very few studies have assessed the effect of acetaldehyde on c-Fos protein expression. Thus, expression of c-Fos mRNA after intravenous administration of a low dose of acetaldehyde was only induced in the paraventricular nuclei of the thalamus (PVTh) (Cao et al., 2007). In another study, blood acetaldehyde accumulated after intraperitoneal (IP) coadministration of EtOH and cyanamide (an ALDH inhibitor; Kinoshita et al., 2002), resulted in a significant increase in c-Fos mRNA in the paraventricular nuclei of the hypothalamus (HPV) (Kinoshita et al., 2002). Thus, it seems that peripheral acetaldehyde accumulation, by direct administration or by blockade of its degradation, results in c-Fos mRNA increases in some brain nuclei (Kinoshita et al., 2002; Cao et al., 2007). However, inhibition of brain catalase activity with aminotriazole did not alter EtOH evoked dose-dependent increases in c-Fos inmunoreactivity in several brain regions (Canales, 2004). This lack of effect after the blockade of centrally generated acetaldehyde could lead to the suggestion that in the brain, only EtOH triggers this neuronal marker. However, no study thus far has investigated the effect of acetaldehyde increases in the brain on c-Fos immunoreactivity.

In the present study we assessed the pattern of c-Fos expression after peripheral (IP) or central (intraventricular, ICV) EtOH and acetaldehyde administration. We analyzed a broad range of prefrontal, basal ganglia and limbic system areas, most of which receive a substantial DA innervation (Fallon and Moore, 1978; Fields, 2007; Ikemoto, 2007), and we used doses of both substances that have been demonstrated to modulate several motor activities and motivated behaviors (Arizzi et al., 2003; Correa et al., 2003a,b; Arizzi-LaFrance et al., 2006; Chuck et al., 2006; McLaughlin et al., 2008) regulated by DA. Peripherally we also administered higher doses of acetaldehyde than the ones used in behavioral studies in order to make additional direct comparisons between EtOH and acetaldehyde.

## Methods

### Subjects

Male Sprague-Dawley rats (290–320 g; *N* = 45) (Harlan Sprague-Dawley, Indianapolis, IN) were housed in a colony maintained at 23°C with a 12 L: 12 D cycle (lights on at 7 h). Water and food were available *ad libitum* in the home cages. In order to minimize the possible effects of receiving a novel potentially stressful injection, rats were handled for 5 days prior to drug administration. For the ICV experiment, the handling was done after recovery from surgery. All animals received humane care in compliance with the protocols approved by the University of Connecticut Institutional Animal Care and Use Committee, and the studies have been conducted according to National Institute of Health Guide for the care and use of animals, National Academy Press (1996) and the EC Directive 86/609/EEC.

### Drugs

EtOH [100%, 200 proof, USP (United States Pharmacopea); AAPER Alcohol and Chemical Co.], acetaldehyde (Fisher Scientific) were dissolved in physiological saline (0.9% w/v) for IP administration and in artificial cerebrospinal fluid (aCSF) for the ICV administration. The aCSF was prepared by mixing sodium chloride, potassium chloride and calcium chloride (147.2 mM NaCl, 2.4 mM CaCl2, 4.0 mM KCl) in purified water. For IP injections, the stock solutions from which the different doses were obtained were: EtOH 20% v/v and acetaldehyde 2% v/v. The doses were 0.5 and 2.5 g/kg of EtOH or 0.1 and 0.5 g/kg acetaldehyde. The two IP doses of EtOH and the lower dose of acetaldehyde were selected based on previous behavioral studies (Chuck et al., 2006; McLaughlin et al., 2008) and the higher dose of acetaldehyde (0.5 g/kg) was selected in order to compare it with the same dose of EtOH. For the ICV studies, EtOH and acetaldehyde were injected at doses of 2.8 and 14.0 μmoles (solutions were 16% and 80% v/v, respectively), in 1.0 μl total volume (EtOH: 129 or 644 μg; Acetaldehyde: 123 or 617 μg). These doses are in the range that had previously produced significant effects in behavioral studies (Arizzi et al., 2003; Correa et al., 2003a,b; Arizzi-LaFrance et al., 2006; McLaughlin et al., 2008). The vehicle control procedure consisted of injections of 1.0 ml of aCSF.

For the surgery, rats were anesthetized with a solution (1.0 ml/kg, IP) that contained ketamine and xylazine (10 ml of 100 mg/ml ketamine plus 0.75 ml of 20 mg/ml xylazine) (Phoenix Pharmaceutical, Inc. St. Joseph, Mo).

### Surgical procedure

For ICV injections, rats were implanted with unilateral guide cannulae (10 mm length 23 ga.). The stereotaxic coordinates for lateral ventricle cannulation were as follows: AP −0.5 mm (from bregma), DL ± 1.3 mm lateral (from midline), and DV −3.0 mm ventral (from the surface of the skull). The incisor bar on the stereotax was set to 0.0 mm above the interaural line. All animals were singly housed after surgery, and were allowed 10 days of recovery. Stainless steel stylets were kept in the guide cannulae to maintain their integrity. Injections were made via 30 ga. stainless steel injectors extending 1.5 mm below the guide cannulae. The injectors were attached to 10.0 ml Hamilton syringes by PE-10 tubing, and were driven by a Harvard Apparatus syringe pump (0.5 ml / min, 1 ml total volume). After the infusions injectors were left in place for 1 min to allow for diffusion of the drug, after which the injectors were removed, stylets were replaced, and animals were placed back into their home cages. The placements of the injectors were verified histologically.

### Tissue processing and c-Fos immunohistochemistry

Animals were anesthetized with CO_2_ and perfused transcardially with 0.9% physiological saline followed by 3.7% paraformaldehyde, 120 min after drug administration. The brains were removed and post-fixed in formalin for 2 days. Thereafter, the brains were cut into three coronal sections, ranging from 3–5 mm in thickness prior to being placed into tissue processing cassettes for paraffin. The tissue cassettes were rinsed in water followed by an EtOH rinse prior to immersion fixation. Paraffin embedded coronal sections were cut (5–7 μm) on a microtome (Leitz Wetzlar, Spencer Scientific Co., New Hampshire) and immediately placed in a 40°C water bath for mounting tissue on Plus slides (Erie Scientific Co, New Hampshire) and allowed to air dry for 24 h. Thereafter, the tissue slides underwent dehydration by a series of three separate washes in citrosolve (2 × 7 min), 100% EtOH (2 × 7 min), and 95% EtOH (2 × 7 min). The slides were rinsed in distilled water and incubated in a 0.3% H_2_O_2_ solution to block endogenous peroxidase activity. The slides were washed (3 × 5 min) in 0.1 M phosphate buffer (PBS) (Dulbecco's phosphate buffered saline; pH 7.4; Sigma Chemical Co) followed by a high pH antigen retrieval (DAKO, Denmark) incubation for 15 min. The slides were then allowed to cool and washed in PBS prior to incubation in the primary antiserum. c-Fos was visualized with a rabbit polyclonal anti-cFos (1:5000, Calbiochem, Germany) for 24 h at room temperature. Following the primary antibody incubation, the sections were washed in PBS (3 × 5 min) and incubated in the secondary, anti-rabbit Horseraddish Peroxidase (HRP)-conjugate envision plus (DAKO, Denmark) for 2 h at room temperature. Thereafter, sections were washed and rinsed for 1–3 min in 3,3′ diaminobenzidine chromagen (DAB) (brown). The sections were then rinsed in distilled water before the hydration series of rinses in 95% EtOH (2 × 7 min), EtOH (2 × 7 min), and citrosolve (2 × 7 min). The slides were cover-slipped using Cytoseal 60 (Thermo Scientific) as a mounting medium and then examined by light microscopy.

### Quantification of c-Fos-labeled cell density

**Figure 1:**
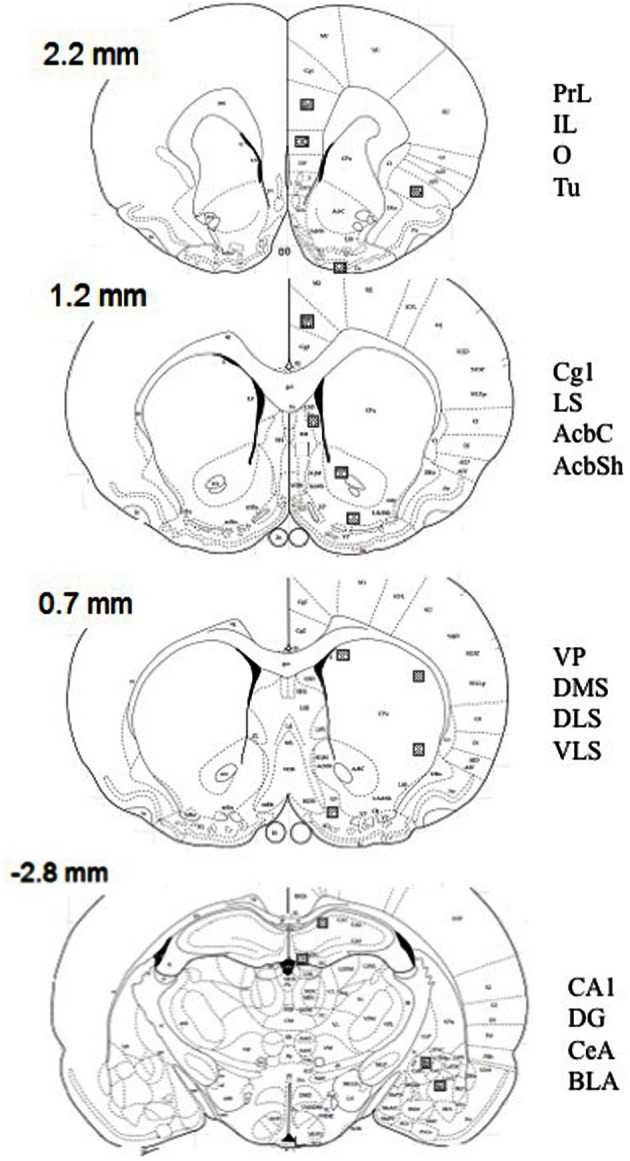
**Schematic diagrams of relevant coronal levels and specific brain regions depicted in Tables 1–6, and Figures 4, 5 [based on the atlas of Paxinos and Watson (1998)].** The squares indicate the placement of optical dissectors for counting c-Fos positive cells. Numbers represent bregma at every coronal level. AcbC, accumbens core; AcbSh, accumbens shell; BLA, basolateral amygdala; CA1, CA1 of the hippocampus; CeA, central amygdala; Cg1, cingulate cortex 1; DG, dentate gyrus; DLS, dorsolateral striatum; DMS, dorsomedial striatum; VLS, ventrolateral striatum; IL, infralimbic cortex; LS, lateral septum; O, orbitofrontal cortex; PrL, prelimbic cortex; Tu, lateral tubercle; VP, ventral pallidum.

Tissue sections were imaged by optic microscopy (Nikon Eclipse E600; Melville, NY) and photographed using SPOT software (Diagnostic Instruments, Inc.). Selected areas of the brain were outlined at low resolution (10×) using known landmarks (see Figure 1 for schematic depictions of regions quantified), and c-Fos-positive cells were identified and quantified at a higher resolution (20×) (0.125 mm^2^/field) by light thresholding. A counting grid (10 × 10) was superimposed on each photomicrograph after background correction. The total density of c-Fos cells were counted by a trained observer, who was unaware of the experimental conditions in a minimum of three adjacent coronal sections. The average value was used for statistical analysis. This manual counting method was validated by comparing results quantified with a modified automated ImageJ software program (v. 1.42, National Institutes of Health sponsored image analysis program) in a total of 20 pictures from different brain areas. The correlation in the scores between both methods was *r* = 0.89 (*p* < 0.01).

### Statistical analysis

For each defined brain region and route of administration, cell counts (c-Fos-positive cells per mm^2^) were compared across treatment groups by means of one way between-groups simple ANOVA with five levels, followed by non-orthogonal planned comparisons using the overall error term, comparing vehicle to the other doses (Keppel, 1991). Significance was set at *p* < 0.05. These analyses were conducted using a computerized statistical package (SPSS). Figures 3, 5 are a representation of these data as a percentage of change in relation to the corresponding vehicle group for every brain structure. No additional statistical analyses were performed.

## Results

Figure 1 shows schematic depictions of brain regions selected. Name and abbreviations are listed in the figure legend.

### Experiment 1. effects of peripheral administration of EtOH and acetaldehyde on c-Fos immunoreactivity in prefrontal cortex (PFC), basal ganglia and limbic areas

The overall one-way ANOVA yielded statistical significance for all the brain areas tested but BLA and DG. These results are depicted in Tables 1–3, respectively. The significance levels for the post hoc analyses are shown in these tables only.

**Table 1:** **Effect of acute IP administration of EtOH or acetaldehyde on c-Fos expression in PFC areas of rat brain**.

**Brain area**	**Vehicle**	**EtOH 0.5 g/kg**	**EtOH 2.5 g/kg**	**Acetal 0.1 g/kg**	**Acetal 0.5 g/kg**
**CELL COUNTS (per mm**^**2**^**) ± SEM**					
Cg1	418.6 ± 47.5	618.4 ± 70.4	678.9 ± 130.3	835.5 ± 129.7^*^	1156.2 ± 125.9^**^^+^^##^
PrL	267.0 ± 22.8	440.8 ± 53.1^*^	303.0 ± 14.8	546 ± 60.8^**^	881.0 ± 49.6^**^^++^^##^
IL	258.0 ± 13.7	372.0 ± 17.7	320.0 ± 16.5	508.8 ± 78.4^**^	787.0 ± 61.9^**^^++^^##^
O	318.0 ± 23.6	589.6 ± 86.3^**^	345 ± 25.9^++^	560.0 ± 63.7^**^	620 ± 38.3^**^

Data are the mean number of c-Fos-positive cells (+SEM) per mm^2^ in the regions listed. (

* p < 0.05,

** p < 0.01 different from vehicle for the same brain region,

+ p < 0.05,

++ p < 0.01 different from the lower dose of the same drug,

## p < 0.01 different from the same dose of EtOH).

**Table 2:** **Effect of acute IP administration of EtOH or acetaldehyde on c-Fos expression in basal ganglia areas of rat brain**.

**Brain area**	**Vehicle**	**EtOH 0.5 g/kg**	**EtOH 2.5 g/kg**	**Acetal 0.1 g/kg**	**Acetal 0.5 g/kg**
**CELL COUNTS (per mm**^**2**^**) ± SEM**					
AcbC	260.0 ± 66.5	486.8 ± 95.7^*^	264.8 ± 25.2^+^	693.7 ± 103.8^**^	858.4 ± 22.8^**^^##^
AcbSh	242.6 ± 51.0	349.5 ± 19.7	325.6 ± 57.8	459.0 ± 55.9^**^	633.2 ± 71.3^**^^+^^##^
VLS	229.3 ± 9.6	456.5 ± 30.8^*^	547.3 ± 95.3^**^	594.1 ± 82.6^**^	648.6 ± 53.5^**^^#^
DLS	258.0 ± 17.2	449.3 ± 19.6^**^	335.4 ± 19.7	569.6 ± 60.1^**^	534.0 ± 50.1^**^
DMS	218.0 ± 29.5	462.4 ± 30.2^**^	405.8 ± 35.6^*^	559.3 ± 91.2^**^	562.0 ± 56.9^**^
VP	151.0 ± 47.0	296.8 ± 23.3^*^	304.0 ± 58.8^*^	328.4 ± 26.1^*^	417.5 ± 70.8^**^^##^

Data are the mean number of c-Fos-positive cells (+SEM) per mm^2^ in the regions listed. (

* p < 0.05,

** p < 0.01 different from vehicle for the same brain region,

+ p < 0.05 different from the lower dose of the same drug,

# p < 0.05,

## p < 0.01 different from the same dose of EtOH).

**Table 3:** **Effect of acute IP administration of EtOH or acetaldehyde on c-Fos expression in limbic areas of rat brain**.

**Brain area**	**Vehicle**	**EtOH 0.5 g/kg**	**EtOH 2.5 g/kg**	**Acetal 0.1 g/kg**	**Acetal 0.5 g/kg**
**CELL COUNTS (per mm**^**2**^**) ± SEM**					
Tu	118.0 ± 8.1	284.0 ± 28.1^**^	244.0 ± 17.5^*^	307.0 ± 36.5^**^	337.0 ± 61.7^**^
LS	124.0 ± 9.3	183.2 ± 18.5	194.4 ± 13.0^*^	263.0 ± 26.9^**^	398.0 ± 41.5^**^^++^^##^
BLA	66.0 ± 3.8	115.2 ± 29.1	44.8 ± 13.5	89.0 ± 14.2	135.0 ± 24.2
CeA	79.0 ± 16.9	118.4 ± 18.6	34.4 ± 16.9^+^	99.0 ± 20.9	172.0 ± 23.2^**^^++^
CA1	74.0 ± 7.3	89.6 ± 7.2	114.4 ± 33.6	79.0 ± 7.7	182 ± 31.4^**^^++^^##^
DG	105.0 ± 15.3	128 ± 18.5	88.0 ± 32.4	90.4 ± 32.8	186.0 ± 38.9

Data are the mean number of c-Fos-positive cells (+SEM) per mm^2^ in the regions listed. (

* p < 0.05,

** p < 0.01 different from vehicle for the same brain region,

+ p < 0.05,

++ p < 0.01 different from the lower dose of the same drug,

## p < 0.01 different from the same dose of EtOH).

Thus, in the PFC the ANOVA results were as follows; Cg1 [*F*_(4, 18)_ = 5.64, *p* < 0.01], PrL [*F*_(4, 17)_ = 24.61, *p* < 0.01], IL [*F*_(4, 17)_ = 16.83, *p* < 0.01], and O [*F*_(4, 17)_ = 5.55, *p* < 0.01]. The planned comparisons indicated that EtOH only induced c-Fos expression in the PrL and O cortices and only at the lowest dose (0.5 g/kg), while both doses of acetaldehyde significantly induced c-Fos in all the cortical areas analyzed. Moreover, acetaldehyde was more efficacious than EtOH in all the cortical areas but O, since 0.5 g/kg acetaldehyde was statistically different from 0.5 g/kg EtOH.

**Figure 2:**
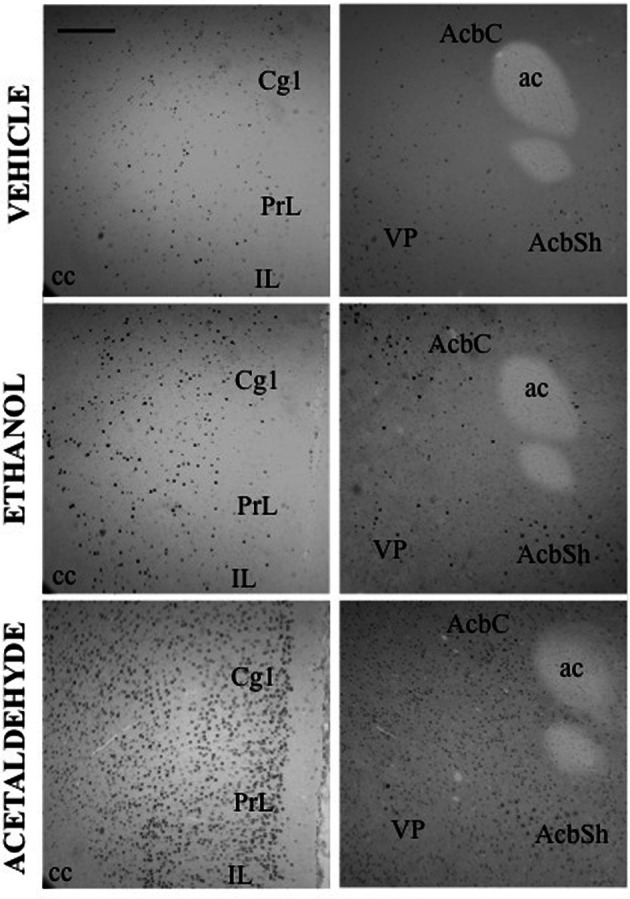
**c-Fos induction after IP administration of saline, ethanol (0.5 g/kg) and acetaldehyde (0.5 g/kg).** Low power images (10×) showing PFC (left column) and Acb (right column). Scale bar = 100 μm. ac, anterior commissure; cc, corpus callosum.

In the basal ganglia structures the overall one-way ANOVA's for all the different regions were significant. The *F* values were as follows: AcbC [*F*_(4, 17)_ = 12.87, *p* < 0.01], AcbSh [*F*_(4, 17)_ = 6.96, *p* < 0.01], VLS [*F*_(4, 17)_ = 6.28, *p* < 0.01], DLS [*F*_(4, 17)_ = 10.94, *p* < 0.01], DMS [*F*_(4, 17)_ = 8.48, *p* < 0.01], and VP [*F*_(4, 17)_ = 4.11, *p* < 0.05]. The planned comparisons indicated that EtOH produced a significant increase at both doses in the VLS, DMS, and VP, while in the AcbC and in the DLS only the low dose induced c-Fos. Surprisingly, none of the EtOH doses induced significantly c-Fos in the AcbSh. Acetaldehyde produced significant induction of c-Fos in all the structures at both doses and it was significantly more efficacious than EtOH at inducing c-Fos in all the ventral areas of the striatum and in the VP, but not in the dorsal areas of striatum (DLS and DMS). Figure 2 shows representative microphotographs of PFC and Acb areas.

As pointed out above, in the limbic areas the one-way ANOVAs were not significant in the BLA and in the DG. However, in the other areas the ANOVAs were significant; Tu [*F*_(4, 17)_ = 5.89, *p* < 0.01], LS [*F*_(4, 17)_ = 8.14, *p* < 0.01], CeA [*F*_(4, 17)_ = 11.48, *p* < 0.01], and CA1 [*F*_(4, 17)_ = 3.66, *p* < 0.05]. EtOH produced a significant effect only in the Tu and in the LS, and these two areas were also more sensitive to the effect of acetaldehyde (i.e., both doses produced an increase).

**Figure 3:**
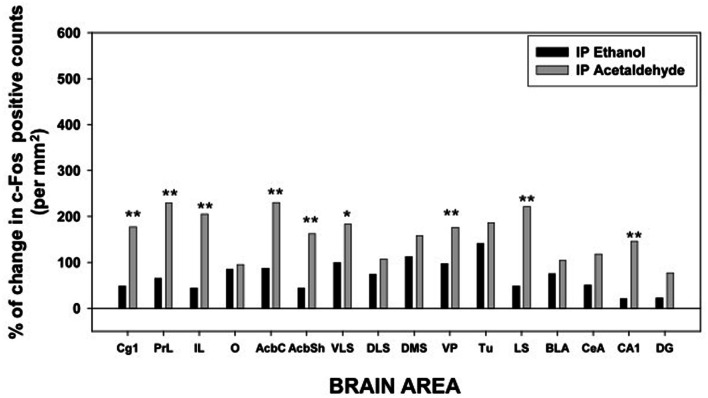
**Percentage of change in c-Fos counts after IP administration of ethanol and acetaldehyde (0.5 g/kg) in relation to the saline vehicle group for every brain structure.** ^*^p < 0.05, ^**^p < 0.01 different from EtOH in that brain area.

Overall, it seems that, among the EtOH groups, while 0.5 g/kg increased c-Fos expression (although it was not always statistically significant), 2.5 g/kg EtOH induced c-Fos only in some areas of the striatum and limbic system, but it did not produce a larger increase than the lower dose, thus possibly indicating a biphasic effect of EtOH on c-Fos expression (see for example PrL and O cortex, AcbC, and CeA). As for the acetaldehyde groups, in general both doses increased c-Fos immunoreactivity at higher levels than the EtOH groups, especially in cortical structures and in both Acb subregions. Acetaldehyde was more efficacious than EtOH at inducing c-Fos immunoreactivity, since EtOH at 0.5 g/kg significantly induced c-Fos in 8 of the 16 areas, while 0.5 g/kg acetaldehyde did so in 14 areas. Moreover, 0.5 g/kg acetaldehyde was significantly different from 0.5 g/kg EtOH in 9 of the 16 areas. The percentage change that this dose produced in relation to vehicle for both drugs in all the structures studied is graphically shown in Figure 3.

### Experiment 2. effects of central administration of EtOH and acetaldehyde on c-Fos immunoreactivity in PFC, basal ganglia and limbic areas

The effects of ICV administration of EtOH or acetaldehyde on c-Fos immunoreactivity are shown in Tables 4–6 for PFC, basal ganglia and limbic areas, respectively. The results of the one-way ANOVA for every brain area showed that the treatment produced significant effects for all the areas studied. Thus in the PFC; Cg1 [*F*_(4, 18)_ = 11.28, *p* < 0.01], PrL [*F*_(4, 19)_ = 10.88, *p* < 0.01], IL [*F*_(4, 19)_ = 15.41, *p* < 0.01], and O [*F*_(4, 19)_ = 19.34, *p* < 0.01]. The planned comparisons indicated that EtOH induced c-Fos only at the low dose (2.8 μmoles) and only in Cg1 and O. However, acetaldehyde produced significant effects in all structures and at both doses. When comparing the low dose (2.8 μmoles) of EtOH with the same dose of acetaldehyde, it was found that only in the Cg1 were there no differences between the two drugs, although the higher dose (14 μmoles) of acetaldehyde induced c-Fos significantly compared with the effect of the high dose of EtOH. In the rest of PFC areas both doses of acetaldehyde produced an increase compared to the corresponding dose of EtOH.

**Table 4:** **Effect of acute ICV administration of EtOH or acetaldehyde on c-Fos expression in PFC areas of rat brain**.

**Brain area**	**Vehicle**	**EtOH 2.8 μmoles**	**EtOH 14 μmoles**	**Acetal 2.8 μmoles**	**Acetal 14 μmoles**
**CELL COUNTS (per mm**^**2**^**) ± SEM**					
Cg1	307.0 ± 48.5	675.0 ± 167.5^*^	356.9 ± 28.2	884.9 ± 169.3^**^	1299.4 ± 131.8^**^^+^^##^
PrL	241.6 ± 20.0	295.2 ± 17.9	257.6 ± 26.0	521.6 ± 43.8^**^^##^	556.0 ± 99.9^**^^##^
IL	244.0 ± 36.0	281.6 ± 28.3	287.2 ± 31.5	539.5 ± 53.6^**^^##^	533.0 ± 19.3^**^^##^
O	318.4 ± 45.3	565.6 ± 50.5^**^	308.0 ± 42.0	610.4 ± 37.2^**^	764.0 ± 28.9^**^^+^^##^

Data are the mean number of c-Fos-positive cells (+SEM) per mm^2^ in the regions listed. (

* p < 0.05,

** p < 0.01 different from vehicle for the same brain region,

+ p < 0.05 different from the lower dose of the same drug,

## p < 0.01 different from the same dose of EtOH).

**Table 5:** **Effect of acute ICV administration of EtOH or acetaldehyde on c-Fos expression in basal ganglia areas of rat brain**.

**Brain area**	**Vehicle**	**EtOH 2.8 μmoles**	**EtOH 14 μmoles**	**Acetal 2.8 μmoles**	**Acetal 14 μmoles**
**CELL COUNTS (per mm**^**2**^**) ± SEM**					
AcbC	78.5 ± 31.5	409.0 ± 96.6^*^	371.0 ± 69.3	504.3 ± 162.1^**^	823.0 ± 150.7^**^^##^
AcbSh	108.4 ± 39.4	377.5 ± 120.9^*^	257.6 ± 60.7	398.5 ± 110.2^*^	503.8 ± 72.6^**^^#^
VLS	372.4 ± 21.7	489.8 ± 72.4	507.6 ± 47.7	616.3 ± 62.1^**^	468.4 ± 48.9
DLS	331.0 ± 15.7	409.4 ± 41.1	426.4 ± 18.1	435.3 ± 75.8	633.2 ± 48.4^**^^++^^##^
DMS	389.6 ± 26.1	366.5 ± 50.4	388.6 ± 48.8	500.1 ± 56.7^**^^#^	691.0 ± 49.9^**^^+^^##^
VP	208.8 ± 9.1	354.0 ± 20.4^**^	360.9 ± 15.1^**^	390.0 ± 40.0^**^	307.0 ± 36.2^*^^+^

Data are the mean number of c-Fos-positive cells (±SEM) per mm^2^ in the regions listed. (

* p < 0.05;

** p < 0.01 different from vehicle for the same brain region,

+ p < 0.05;

++ p < 0.01 different from the lower dose of the same drug,

# p < 0.05,

## p < 0.01 different from the same dose of EtOH).

**Table 6:** **Effect of acute ICV administration of EtOH or acetaldehyde on c-Fos expression in limbic areas of rat brain**.

**Brain area**	**Vehicle**	**EtOH 2.8 μmoles**	**EtOH 14 μmoles**	**Acetal 2.8 μmoles**	**Acetal 14 μmoles**
**CELL COUNTS (per mm**^**2**^**) ± SEM**					
Tu	214.4 ± 27.2	369.6 ± 55.7^*^	241.6 ± 34.4^+^	425.6 ± 27.9^**^	509.0 ± 46.6^**^^##^
LS	224.8 ± 28.6	248.0 ± 22.0	214.4 ± 13.0	325.6 ± 29.7^*^^#^	446.0 ± 31.4^**^^++^^##^
BLA	72.0 ± 6.8	144.0 ± 20.2^*^	196.0 ± 13.2^**^	187.0 ± 20.0^*^	146.0 ± 40.0^**^
CeA	60.8 ± 13.7	142.4 ± 27.1	238.4 ± 20.8^**^^+^	193.6 ± 32.2^**^	125.0 ± 53.5^#^
CA1	91.2 ± 8.6	196.8 ± 12.2^**^	227.2 ± 13.1^**^	160.8 ± 31.2	259.0 ± 57.0^**^^+^
DG	89.6 ± 8.5	141.6 ± 14.8	243.2 ± 14.6^**^^++^	199.2 ± 29.0^**^	168.0 ± 52.4^*^

Data are the mean number of c-Fos-positive cells (±SEM) per mm^2^ in the regions listed. (

* p < 0.05,

** p < 0.01 different from vehicle for the same brain region,

+ p < 0.05,

++ p < 0.01 different from the lower dose of the same drug,

# p < 0.05,

## p < 0.01 different from the same dose of EtOH).

**Figure 4:**
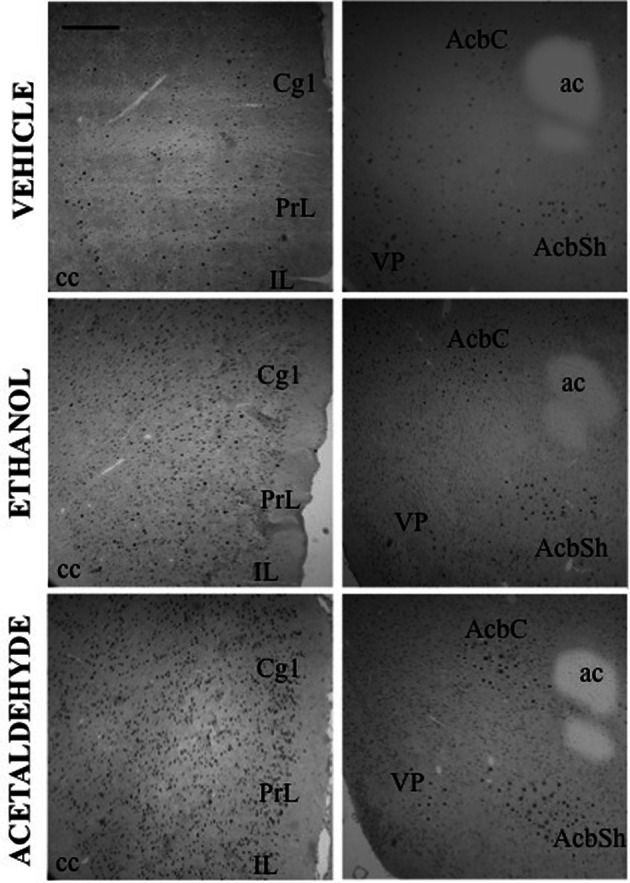
**c-Fos induction after ICV administration of vehicle, ethanol (2.8 μmoles) and acetaldehyde (2.8 μmoles).** Low power images (10×) showing PFC (left column) and Acb (right column). Scale bar = 100 mm.

The differences between ETOH and acetaldehyde were not so pronounced in the basal ganglia or limbic structures. The one-way ANOVA for the different structures indicated that the treatment had a significant overall effect in all of them. The *F* values were as follows: AcbC [*F*_(4, 18)_ = 5.61, *p* < 0.01], in the AcbSh [*F*_(4, 18)_ = 3.24, *p* < 0.05], in the VLS [*F*_(4, 17)_ = 3.12, *p* < 0.05], in the DLS [*F*_(4, 18)_ = 5.16, *p* < 0.01], in the DMS [*F*_(4, 18)_ = 7.45, *p* < 0.01], and in the VP [*F*_(4, 17)_ = 7.26, *p* < 0.01]. The planned comparisons indicated that the low dose of EtOH significantly induced c-Fos in both subregions of the Acb and also in the projection area VP. However, the high dose of EtOH produced an increase only in the VP. The low dose of acetaldehyde induced c-Fos in the ventral areas of the striatum (including both Acb subregions and VLS) and in the VP, but not in the dorsal striatum. However there were no differences between EtOH and acetaldehyde at this behaviorally relevant dose. Differences between EtOH and acetaldehyde emerged only at the highest dose in both subregions of the Acb and in both areas of the dorsal striatum. See representative microphotographs of PFC and Acb areas in Figure 4.

**Figure 5:**
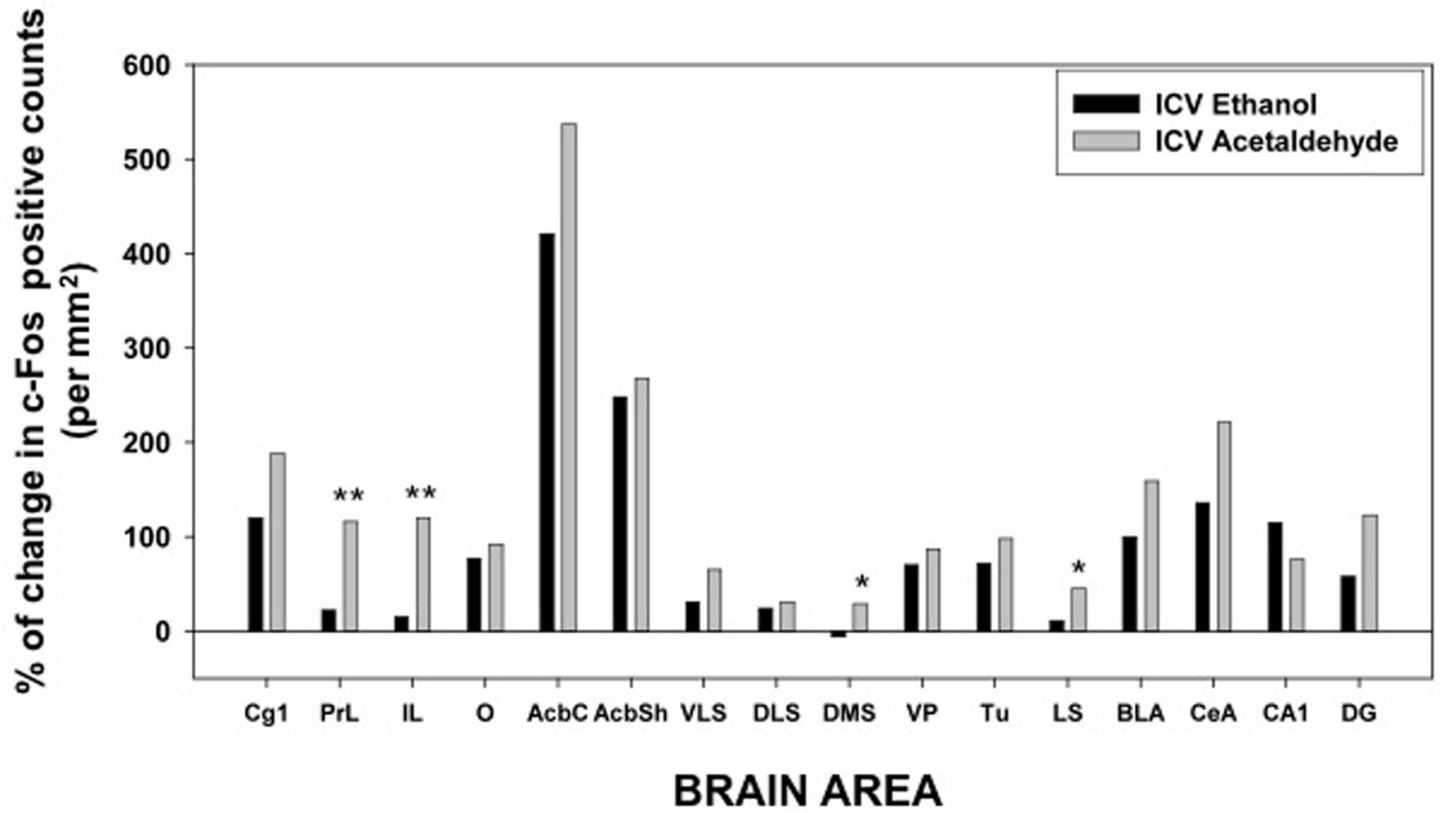
**Percentage of change in c-Fos counts after ICV administration of ethanol and acetaldehyde (2.8 μmoles) in relation to the aCSF vehicle group for every brain structure.** ^*^p < 0.05, ^**^p < 0.01 different from EtOH in that brain area.

The one-way ANOVA for the limbic regions also demonstrated a significant overall effect of the treatment in all areas studied. The F values were as follows: Tu [*F*_(4, 19)_ = 9.39, *p* < 0.01], LS [*F*_(4, 19)_ = 13.01, *p* < 0.01], BLA [*F*_(4, 19)_ = 5.72, *p* < 0.01], CeA [*F*_(4, 19)_ = 5.23, *p* < 0.01], CA1 [*F*_(4, 19)_ = 5.60, *p* < 0.01], and DG [*F*_(4, 19)_ = 5.16, *p* < 0.01]. The planned comparisons demonstrated that the low dose of EtOH produced significant increases only in three of the areas (Tu, BLA, and CA1), while the higher dose did so in both areas of the amygdala and of the hippocampus. Acetaldehyde produced a more robust increase, since at the low dose all areas but CA1 showed increased c-Fos immunoreactivity compared to vehicle. At the high dose all areas but CeA showed increased c-Fos counts. Significant differences between both drugs were only seen in the Tu and in the LS at both doses, while in the CeA the high dose of EtOH produced an increase in c-Fos that was significantly different from acetaldehyde, which at the highest dose did not significantly induced c-Fos compared to vehicle. The percentage change that 2.8 μmoles of EtOH and acetaldehyde produced in relation to vehicle is graphically shown in Figure 5.

## Discussion

The purpose of the present study was twofold: first, to compare the pattern of c-Fos induction after EtOH and its metabolite acetaldehyde were administered by two routes of administration that have been demonstrated to reveal differences in the potency between both drugs, and second, we chose to study brain areas with DArgic innervations because both drugs have demonstrated to have effects on motor activity and motivated behaviors regulated by DA. Thus, comparisons between behaviorally relevant doses of both compounds after both routes of administration revealed the impact of each drug on different areas of the brain.

### Effects of peripheral administration of ethanol and acetaldehyde

Overall, control values (saline or aCSF treatments) reflected comparable levels of c-Fos across the different routes of administration for all 16 brain regions quantified. In no case did EtOH or acetaldehyde significantly reduce basal c-Fos levels. Peripheral administration was the route that generated higher differences between EtOH and acetaldehyde at the same dose. However, ICV administration showed more contrasting effects between brain areas, though less so between drugs. These results reflect the same pattern of results found in behavioral studies (Arizzi et al., 2003; Correa et al., 2003a,b; Arizzi-LaFrance et al., 2006; Chuck et al., 2006; McLaughlin et al., 2008).

Thus, as it can be seen in Figure 3, with the exception of the O, PFC areas were significantly more responsive to peripherally administered acetaldehyde than to EtOH (see pictures in Figure 2). In Figure 3 it can also be appreciated that the magnitude of change in c-Fos counts from vehicle at this dose was significantly bigger for acetaldehyde in ventral basal ganglia structures. However, these differences were less robust in limbic structures, in which only LS and CA1 revealed a significantly larger impact of acetaldehyde. Very few previous studies have addressed the impact of peripheral acetaldehyde, either locally formed or exogenously administered, on c-Fos immunoreactivity. Expression of *c-Fos* mRNA in the HPV was increased after peripheral accumulation of acetaldehyde by blocking ALDH activity with cyanamide and administering a dose of EtOH (1 g/kg) that did not have this effect on its own (Kinoshita et al., 2002). Kinoshita et al. (2002) did not explore any other brain area, thus further comparisons with the present results are not possible. So far, there is only one previous study of direct peripheral administration of acetaldehyde and c-Fos expression (Cao et al., 2007). In that study a small dose of acetaldehyde (0.032 g/kg) was administered also to Sprague-Dawley rats using intravenous injections as the peripheral route of administration. In agreement with the results of the 0.1 g/kg in the present study, 0.032 g/kg did not produce a significant increase in the CeA, but in contrast to our results, it did not induce c-Fos in the AcbSh (Cao et al., 2007). That dose was only able to increase c-Fos mRNA in the PVTh, possibly because this dorsal thalamic region is minimally protected by the blood brain barrier (Ueno et al., 2000; Cao et al., 2007). Moreover, the dose of acetaldehyde used in that study seems to be very low, since it did not affect ambulation or anxiety parameters (Cao et al., 2007). A dose of acetaldehyde of 0.1 g/kg IP, like the lowest one used in the present study, was demonstrated to induce anxiety and reduce locomotion in mice (Tambour et al., 2005; Escrig et al., 2012), and to slow lever pressing in rats (McLaughlin et al., 2008), although in this last study, higher doses of acetaldehyde (0.2 g/kg) were necessary to produce a significant suppression of total lever pressing. For EtOH, the required doses to slow lever pressing performance and to suppress total amount of lever pressing were 0.8 and 1.6 g/kg (McLaughlin et al., 2008), indicating that the two drugs also show differences in potency in terms of their behavioral effects.

### Effects after central administration of ethanol and acetaldehyde

After central administration, only 4 brain areas showed significant differences between EtOH and acetaldehyde at the lowest dose of both drugs (2.8 μmoles): two cortical structures, PrL and IL (see pictures in Figure 4), DMS and LS. The highest dose (14 μmoles) of EtOH and acetaldehyde was the one that lead to major differences between drugs after ICV administration (11 out of 16 structures). Interestingly, EtOH seems to show a biphasic effect at this dose since in many structures EtOH did not differ from vehicle. Acetaldehyde at the high dose had a very different pattern because, in all but two structures, there was still a significant increase in expression of c-Fos compared to vehicle, and in several cases these changes were even bigger than the ones produced by the lowest dose of acetaldehyde. Similar high doses have been used in previous studies (Arizzi et al., 2003; Crankshaw et al., 2003) in which a dose of 17.6 μmoles of EtOH and acetaldehyde did not produce a significant change in operant responding for food compared to vehicle in a task that required minimal rates of responding, while acetate, a metabolite of acetaldehyde, did suppress behavior at these dose. Moreover, a dose of around 17 μmoles of EtOH administered ICV did not produce conditioned taste aversion but it induced conditioned taste preference to a sweet solution (Crankshaw et al., 2003). These data indicate that although it is a high dose, the present 14 μmoles dose does not affect behavioral outcomes such as sustained attention, lever pressing, eating, or taste related learning (Arizzi et al., 2003; Crankshaw et al., 2003).

Two studies so far have measured c-Fos expression after ICV EtOH administration in some areas of the brain (Crankshaw et al., 2003; Larkin et al., 2010). Both of them have used higher doses (790 μg and 4 mg; Crankshaw et al., 2003; Larkin et al., 2010) than the high dose used in the present study (14 μmoles = 644 μg). The pattern of results found by those studies was different from the present ones: positive increases in Cg cortex, (Larkin et al., 2010), AcbSh and LS (Crankshaw et al., 2003), and no increase in the CeA (Crankshaw et al., 2003; Larkin et al., 2010). No previous study has assessed the involvement of acetaldehyde in c-Fos expression after central administration. However, catalase activity inhibition by aminotriazole (thus, blockade of brain acetaldehyde formation), did not affect c-Fos expression in Acb and CeA, after peripheral EtOH administration (Canales, 2004). Those results point to an independent effect of EtOH on this cellular parameter of activity.

### Impact of ethanol and acetaldehyde on Acb-DA related functions

Among all the structures studied in the present work, the Acb warrants additional examination. AcbC was revealed as the more sensitive area to c-Fos induction after EtOH or acetaldehyde with both routes of administration (see pictures in Figures 2, 4). After peripheral administration, EtOH 0.5 g/kg produced a significant induction of c-Fos immunoreactivity in AcbC, although not in AcbSh. These results are somehow consistent with previous studies from other laboratories. For example, after IP administration of 1.0 or 2.5 g/kg of EtOH, also in Sprague-Dawley rats, there was an increase on c-Fos expression in the general area of the Acb (Canales, 2004). However, although in the present study the increase in the AcbC was seen after the administration of a smaller dose (0.5 g/kg), we did not find a significant increase at 2.5 g/kg, thus possibly pointing to a typical biphasic effect of EtOH in this type of cellular markers in the Acb (Ibba et al., 2009). In other studies c-Fos expression after 2.5 g/kg IP of EtOH, did not produce an effect on AcbC but it did significantly induced c-Fos in AcbSh (47% increase) (Knapp et al., 2001). In the present study 2.5 g/kg EtOH tended to induce c-Fos in the AcbSh to a very similar magnitude (34%), although this difference was not statistically significant. On the other hand, after central administration of 2.8 μmoles (129 μg) of EtOH the increases in c-Fos expression produced in both Acb subregions (421% for the AcbC and 248% for AcbSh) reached statistical significance (see Figure 5). In the only study of c-Fos expression after ICV EtOH administration that analyzed the Acb area (Crankshaw et al., 2003), it was found a significant increase (127%) in the AcbSh after a single injection of 790 μg of EtOH. In our study, the highest dose (644 μg) of EtOH did not produce a statistically significant increase, although the percentage of increase from vehicle was 375% for the AcbC and 138% for the AcbSh, an increase very similar to the above mentioned study. The effects of acetaldehyde in the Acb showed a significant increase at both doses and after both routes of administration in both subregions (see pictures in Figures 2, 4). Both doses of acetaldehyde increased c-Fos expression significantly in AcbC (166 and 230%) and in AcbSh (89 and 161%). Moreover, 0.5 g/kg acetaldehyde produced a significantly higher increase than the same dose of EtOH both in AcbC and in AcbSh (see Figure 3). After central administration of acetaldehyde the pattern of effects was the same as for peripheral administration. There was a difference in efficacy at the highest dose; 14 μmoles of acetaldehyde, both in the AcbC and in the AcbSh, increased c-Fos expression while 14 μmoles of EtOH did not produce a significant change. However, there was no difference in efficacy between EtOH and acetaldehyde at a more behaviorally relevant dose, 2.8 μmoles (see Figure 5), as it was the case in food reinforced lever pressing (Arizzi et al., 2003; McLaughlin et al., 2008).

EtOH- and acetaldehyde-induced changes in c-Fos expression of DA target areas may be mediated by modulation of DA release and DA receptor activation. Acetaldehyde has been demonstrated to induce neuronal firing of DArgic neurons in the ventral tegmental area (Foddai et al., 2004; Diana et al., 2008) and to stimulate DA transmission in the Acb (Melis et al., 2007; Enrico et al., 2009; Sirca et al., 2011; Deehan et al., 2013). Extracellular regulated kinase (ERK) activation has been suggested as a postsynaptic correlate of activated DA transmission (Acquas et al., 2007), and acetaldehyde has been reported to elicit ERK phosphorylation in the rat Acb and extended amygdala, via DA D1 receptors (Vinci et al., 2010; Peana et al., 2011). Thus, peripheral intragrastric administration of EtOH (0.5–2.0 g/kg) increased pERK in the AcbC and AcbS in a biphasic dose response way (Ibba et al., 2009). A much lower dose of acetaldehyde (0.02 g/kg) induced ERK phosphorylation also in AcbC, AcbSh (Vinci et al., 2010; Peana et al., 2011). This is also important because ERK seems to be necessary for the induction of c-Fos expression after alcohol administration (Bachtell et al., 2002).

## Conclusion

From the present study we can conclude that EtOH and acetaldehyde produce a general pattern of c-Fos induction in PFC, basal ganglia, and limbic areas, most of which have a substantial DA innervation (Fields, 2007; Ikemoto, 2007), at doses that are able to affect motor activity and motivated behaviors (Arizzi et al., 2003; Correa et al., 2003a,b; Arizzi-LaFrance et al., 2006; Chuck et al., 2006; McLaughlin et al., 2008). Peripherally administered acetaldehyde is more potent than EtOH at suppressing motor behaviors (McLaughlin et al., 2008), and the same potency pattern can be seen in relation to c-Fos expression (present paper). The present results are also in accordance with the behavioral data on locomotion and lever pressing after central administration. Both drugs do not show big differences in efficacy or potency when injected in the ventricles (Arizzi et al., 2003; Correa et al., 2003a; Arizzi-LaFrance et al., 2006; McLaughlin et al., 2008). Thus, c-Fos can be used as a general marker of neural activity in DA terminal areas, one which reflects the impact of EtOH and acetaldehyde after different routes of administration on motor and motivational functions.

### Conflict of interest statement

The authors declare that the research was conducted in the absence of any commercial or financial relationships that could be construed as a potential conflict of interest.

## Abbreviations

AcbC: accumbens core
AcbSh: accumbens shell
ADH: alcohol dehydrogenase
ALDH: aldehyde dehydrogenase
aCSF: artificial cerebrospinal fluid
ac: anterior commissure
BLA: basolateral amygdala
CA1: CA1 of the hippocampus
CeA: central amygdala
Cg1: cingulate cortex 1
cc: corpus callosum
DG: dentate gyrus
DA: dopamine
DLS: dorsolateral striatum
DMS: dorsomedial striatum
EtOH: ethanol
ERK: extracellular regulated kinase
VLS: ventrolateral striatum
IL: infralimbic cortex
IP: intraperitoneal
ICV: intraventricular
LS: lateral septum
O: orbitofrontal cortex
HPV: paraventricular nuclei of the hypothalamus
PVTh: paraventricular nuclei of the thalamus
PrL: prelimbic cortex
Tu: lateral tubercle
VP: ventral pallidum.
